# Density Functional Theory Insight in Photocatalytic Degradation of Dichlorvos Using Covalent Triazine Frameworks Modified by Various Oxygen-Containing Acid Groups

**DOI:** 10.3390/toxics12120928

**Published:** 2024-12-21

**Authors:** Shouxi Yu, Zhongliao Wang

**Affiliations:** Anhui Province Industrial Generic Technology Research Center for Alumics Materials, Anhui Province Key Laboratory of Pollutant Sensitive Materials and Environmental Remediation, School of Physics and Electronic Information, Huaibei Normal University, Huaibei 235000, China; chnu19810901912@163.com

**Keywords:** organophosphate pesticide, photocatalytic degradation, covalent triazine frameworks, oxygen-containing acid groups, DFT calculation

## Abstract

Dichlorvos (2,2-dichlorovinyl dimethyl phosphate, DDVP) is a highly toxic organophosphorus insecticide, and its persistence in air, water, and soil poses potential threats to human health and ecosystems. Covalent triazine frameworks (CTFs), with their sufficient visible-light harvesting capacity, ameliorated charge separation, and exceptional redox ability, have emerged as promising candidates for the photocatalytic degradation of DDVP. Nevertheless, pure CTFs lack effective oxidative active sites, resulting in elevated reaction energy barriers during the photodegradation of DDVP. In this work, density functional theory (DFT) calculations were employed to investigate the impact of various oxygen-containing acid groups (-COOH, -HSO_3_, -H_2_PO_3_) on DDVP photodegradation performance. First, simulations of the structure and optical properties of modified CTFs reveal that oxygen-containing acid groups induce surface distortion and result in a redshift in the absorption edge. Subsequently, analysis of the density of states, frontier molecular orbitals, surface electrostatic potential, work function, and dipole moment demonstrates that oxygen-containing acid groups enhance CTF polarization, facilitate charge separation, and ameliorate their oxidative capability. Additionally, the free-energy diagram of DDVP degradation uncovers that oxygen-containing acid groups lower the energy barrier by elevating the adsorption and activation capability of DDVP. Notably, -H_2_PO_3_ presents optimal potential for the photodegradation of DDVP by unique electronic structure and activation capability. This work offers a valuable reference for the development of oxygen-containing acid CTF-based photocatalysts applied in degrading toxic organophosphate pesticides.

## 1. Introduction

Dichlorvos (2,2-dichlorovinyl dimethyl phosphate, DDVP) is a highly effective organophosphorus insecticide extensively applied in agriculture to control various pests. However, due to its high toxicity and potential risks to ecosystems and human health, it has emerged as a critical environmental pollutant of concern. Current remediation strategies for DDVP include chemical degradation, microbial degradation [1], and environmental degradation. However, high costs, inefficiency, or dependency on environmental conditions limit their practical application [2]. In contrast, photocatalysis offers an eco-friendly, efficient, and economical channel for the sustainable degradation of DDVP. Traditional catalysts, such as ZnO [3], BiVO_4_ [4], g-C_3_N_4_ [5], WO_3_ [6]_,_ MnO_2_ [7], et al., face challenges in effectively degrading DDVP due to severe electron-hole recombination, inefficient light harvesting, complex surface structure, and ambiguous active sites. Covalent triazine frameworks (CTFs) comprise donor-acceptor groups that facilitate fast charge separation, rendering them promising candidates for degrading DDVP [8,9,10]. Additionally, CTFs possess a relatively simple and well-defined structure with clearly identifiable active sites, providing an ideal platform for theoretical studies and mechanistic exploration [11,12]. However, the insufficient active sites and weak affinity capability lead to difficulty in the adsorption and activation of DDVP and further trigger a high degradation energy barrier [13]. Therefore, endowing CTFs with abundant active sites by tailored modification remains imperative.

Photodegradation reaction primarily relies on oxidation processes, where the oxidation capacity of active sites directly influences the degradation efficiency of the target pollutants [14]. Therefore, the development of active sites with high oxidation capabilities is crucial for enhancing photodegradation performance. However, oxidation sites are susceptible to photo-corrosion during photodegradation reactions, which can result in structural damage and performance deterioration. To achieve more efficient and durable photodegradation, it is imperative to enhance the oxidation capacity and the chemical stability of these active sites [15]. Due to their robust antioxidant properties, oxygen-containing acid functional groups exhibit distinct advantages for DDVP photodegradation, particularly by mitigating photo-corrosion and self-degradation under light irradiation. During the process of photodegradation, when a catalyst absorbs light energy, it triggers the excitation of electrons, causing them to transition from the valence band to the conduction band. Simultaneously, this process leads to the accumulation of photogenerated holes within the valence band. Owing to their greater electronegativity, photoexcited holes tend to accumulate in the oxygen-containing acid groups. Therefore, oxygen-containing acid groups can function as active sites for DDVP photodegradation. In addition, the presence of oxygen-rich acid groups induces an asymmetrical charge distribution and a robust dipole moment, thereby expediting the separation of electron-hole pairs. Consequently, the incorporation of appropriate oxygen-containing acid functional groups into CTFs is crucial for enhancing the photocatalytic degradation efficiency of DDVP [16].

Therefore, several common and stable oxygen-containing acid functional groups, such as COOH (carboxyl), HSO_3_ (sulfonic), and H_2_PO_3_ (phosphoric) groups, were selected for the modification of CTFs. The X-ray diffraction (XRD), Fourier-transform infrared (FTIR) spectroscopy, and Raman spectra were simulated by theoretical calculations to examine the changes in structural and optical properties induced by oxygen-containing acid functional groups. Additionally, the influence of these functional groups on the light absorption properties and electronic structure of CTF was examined through ultraviolet-visible (UV–Vis) spectroscopy and density of states (DOS) calculations. Calculations of the dipole moment and surface electrostatic potential were conducted to further investigate charge separation efficiency and the distribution of active sites. Ultimately, the rate-determining step and reaction barriers for DDVP degradation were analyzed by calculating the reaction-free energy profile. These theoretical studies elucidated the reaction mechanism and underscored the potential advantages of oxygen-containing acid functional CTFs in DDVP photodegradation.

## 2. Computational Details

The analysis of spectroscopic properties, including vibrational modes and high-energy states, was performed using CP2K (version 2024.2), applying the PBE functional to ensure a consistent description of the systems. Electronic structure calculations were conducted within the unrestricted Kohn-Sham formalism, utilizing the Gaussian and plane waves (GPW) approach, with Goedecker-Teter-Hutter (GTH) pseudopotentials and a TZV2P-MOLOPT-GTH basis set for all elements. Computational parameters included a plane-wave cutoff of 410 Ry, an SCF convergence criterion of 5 × 10^−6^ hartree, and force convergence set to 5 × 10^−4^ Bohr per hartree. A vacuum layer of 20 Å was introduced to isolate the periodic slabs and minimize surface interactions. For capturing Van der Waals (vdW) forces, the DFT-D3 method with Grimme’s zero-damping function was applied. The Gibbs free energy changes were computed using the equation Δ*G* = Δ*E* + Δ*E*_ZPE_ − *T*Δ*S*, where Δ*E* represents the electronic energy variation, Δ*E*_ZPE_ is the change in zero-point energy, and Δ*S* accounts for the temperature-dependent entropy variation. Zero-point energy corrections and vibrational frequencies, alongside entropy calculations for gaseous molecules, were evaluated using the Shermo software (version 2.6) [17]. Excited-state analysis and spectral studies were performed using Multiwfn software (version 3.8(dev)) [18].

## 3. Results and Discussion

**Figure 1:**
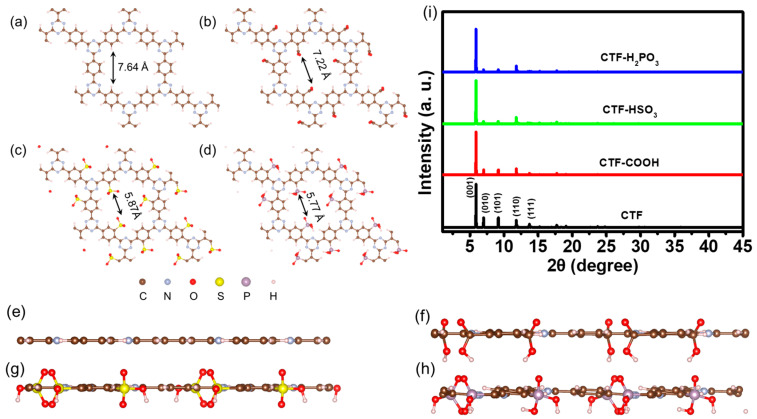
Top and side perspective of crystal structures: (**a**,**e**) CTF, (**b**,**f**) CTF-COOH, (**c**,**g**) CTF-HSO_3_, and (**d**,**h**) CTF-H_2_PO_3_. Simulated (**i**) XRD pattern.

Covalent triazine frameworks, characterized by their distinctive alternating arrangement of triazine (TR) and pyridine rings (Figure 1a), have emerged as a hotspot due to their versatile structural and functional properties. Therefore, single-layer CTFs incorporating various acidic functional groups (-COOH, -HSO_3_, and -H_2_PO_3_) were engineered, which were named CTF-COOH, CTF-HSO_3_, and CTF-H_2_PO_3_. Notably, the acidic functional groups are directly linked to the TR. The optimized structure illustrates that attaching different acidic functional groups to CTF markedly decreases its pore size. As depicted in Figure 1a–h, pristine CTF possesses the largest pore size of 7.64 Å. Upon functionalization, the pore sizes of CTF-COOH, CTF-HSO_3_, and CTF-H_2_PO_3_ are reduced to 7.22 Å, 5.87 Å, and 5.77 Å, respectively, indicating that the incorporation of acidic groups effectively modifies the pore dimensions of CTF. Simulated X-ray diffraction (XRD) results indicate that the characteristic diffraction peaks of CTFs primarily fall within the 5–15° range (Figure 1i). Specifically, pristine CTF shows five distinct peaks at 5.89°, 7.00°, 9.15°, 11.79°, and 13.73°, which are attributed to the (001), (010), (101), (110), and (111) crystal planes, respectively. Notably, the XRD patterns of the functionalized derivatives, CTF-COOH, CTF-HSO_3_, and CTF-H_2_PO_3_, exhibit the same diffraction peaks as the pristine CTF, suggesting that the introduction of acidic functional groups does not significantly affect the lattice parameters. This observation strongly suggests that the ordered structure of the CTF framework is well preserved after functionalization. Further analysis of the simulated structures for CTF-COOH, CTF-HSO_3_, and CTF-H_2_PO_3_ reveals that their lattice parameters remain largely unchanged compared to pristine CTF. The lattice parameters for CTF-COOH were determined to be a = 14.56 Å, b = 14.55 Å, c = 15.00 Å; for CTF-HSO_3_, a = b = 14.58 Å, c = 15.00 Å; and for CTF-H_2_PO_3_, a = 14.60 Å, b = 14.62 Å, c = 15.00 Å. These values are nearly identical to the lattice parameters of pristine CTF (a = b = 14.57 Å, c = 15.00 Å). The results suggest that the incorporation of functional groups effectively preserves the structural integrity of the CTF framework. This consistent crystalline arrangement highlights the robust nature of the CTF material, demonstrating its capacity to accommodate diverse functional groups while maintaining its ordered crystal structure and overall stability.

**Figure 2:**
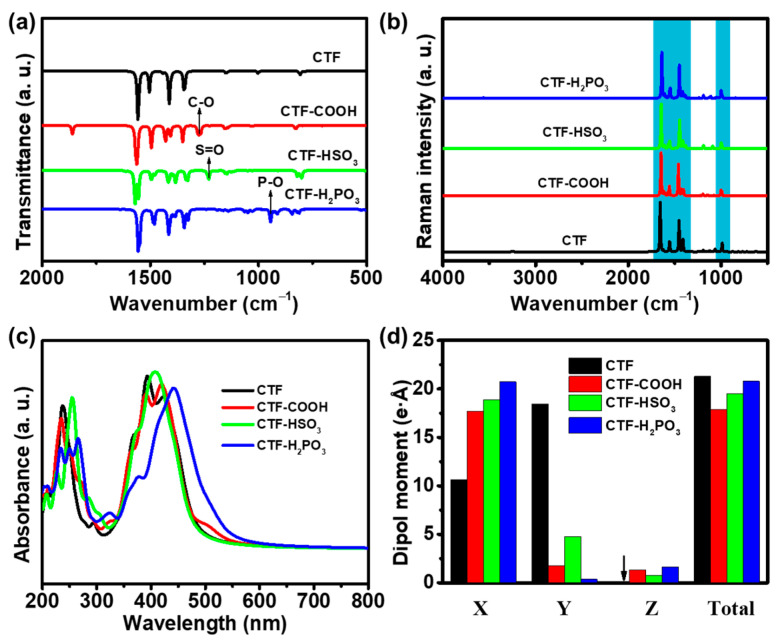
(**a**) IR spectra, (**b**) Raman spectra, and (**c**) UV–Vis spectra of CTF, CTF-COOH, CTF-HSO_3_, and CTF-H_2_PO_3_. (**d**) Dipole moments on different components of CTF, CTF-COOH, CTF-HSO_3_, and CTF-H_2_PO_3_.

Infrared spectroscopy identifies vibrational frequencies and related parameters, offering insights into molecular interactions with their functional groups. As illustrated in Figure 2a, the infrared spectra of CTF, CTF-COOH, CTF-HSO_3_, and CTF-H_2_PO_3_ were simulated within the 500–4000 cm^−1^ range. The CTF exhibits prominent infrared absorption peaks at 1412 and 1504 cm^−1^. For the functionalized variants, CTF-COOH shows peaks at 1407 and 1495 cm^−1^, CTF-HSO_3_ at 1416 and 1496 cm^−1^, and CTF-H_2_PO_3_ at 1415 and 1487 cm^−1^, corresponding to the characteristic infrared absorption of the TR units [11]. Interestingly, further analysis of the spectra reveals additional peaks that are specific to the functional groups introduced. For instance, CTF-COOH displays a distinct absorption peak at 1272 cm^−1^, which is absent in the pure CTF structure. This peak corresponds to the C-O stretch in the -COOH group, confirming the successful incorporation of the -COOH functional group into the CTF framework [19]. Similarly, CTF-HSO_3_ presents a peak at 1229 cm^−1^, attributable to the S=O stretching vibration in the -HSO_3_ group [20], indicating its presence within the CTF structure. In addition, CTF-H_2_PO_3_ shows a characteristic peak at 944 cm^−1^, corresponding to the P-O stretching vibration in the group [21], further corroborating the functionalization of CTF with this group. To further elucidate the crystal structure of CTF, Raman spectra (Figure 2b) simulations were conducted. The results revealed that the benzene rings within CTF exhibit four characteristic absorption peaks at 1453, 1556, 1623, and 1656 cm^−1^, corresponding to the stretching vibrations of the benzene ring [22]. These peaks were similarly observed in the CTF-COOH, CTF-HSO_3_, and CTF-H_2_PO_3_ crystals, though with a redshift. This redshift can be attributed to the influence of the functional groups. Furthermore, the C-H stretching peaks for CTF-COOH, CTF-HSO_3_, and CTF-H_2_PO_3_ were observed at 1002, 991, and 991 cm^−1^, respectively, indicating a blue shift relative to the peak at 988 cm^−1^ in the CTF sample. This blueshift is likely due to the interaction between the attached functional groups and the CTF framework, suggesting that the incorporation of functional groups induces changes in the electronic environment and alters the C-H bond vibrations within the material. This observation further emphasizes the impact of functionalization on the vibrational properties of the CTF structure.

To explore how the attachment of functional groups influences the optical absorption properties of CTFs, the UV–Vis absorption spectra simulated by electronic transition calculations were performed (Figure 2c). In its unmodified form, CTF displayed two absorption peaks: one in the 220–260 nm range, attributed to intramolecular electron transfer [11], and another in the 300–450 nm region, associated with electronic excitation processes [16]. The absorption edge of CTF is observed to be approximately 480 nm, indicating a limited capacity for visible light absorption. The absorption spectrum of CTF was expanded from 480 nm to 550 nm through functionalization with various groups. Among the functionalized CTFs, CTF-H_2_PO_3_ showed the largest shift, with an absorption edge at 550 nm. This enhanced absorption is likely due to the interactions between the functional groups and the CTF structure, which can modify the electronic energy levels of CTF. These interactions lower the energy required for electronic transitions, leading to an expanded absorption range. The extension of the absorption range into the visible region suggests that the functional groups modify the electronic structure of CTF, enhancing its photodegradation potential.

To further investigate the charge distribution after introducing oxygen-containing groups, dipole moment calculations for CTF, CTF-COOH, CTF-HSO_3_, and CTF-H_2_PO_3_ (Figure 2d) were performed. The dipole moment is a crucial indicator of molecule interaction with light, particularly in the visible and near-infrared regions, which is important for photocatalytic performance. Notably, while the dipole moments of CTF-COOH, CTF-HSO_3_, and CTF-H_2_PO_3_ in the X-direction are significantly larger than that of CTF, the overall dipole moments across all four materials are similar. This indicates that the functionalization does not significantly alter the absorption edge width, which is consistent with the trends observed in the UV–Vis absorption spectra. Furthermore, the dipole moment is also indicative of the internal charge distribution, with CTF-H_2_PO_3_ exhibiting the highest dipole moment in the X-direction. This suggests the presence of the strongest built-in electric field in CTF-H_2_PO_3_, which is likely to facilitate the efficient separation of photogenerated charge carriers, thus improving photocatalytic performance.

**Figure 3:**
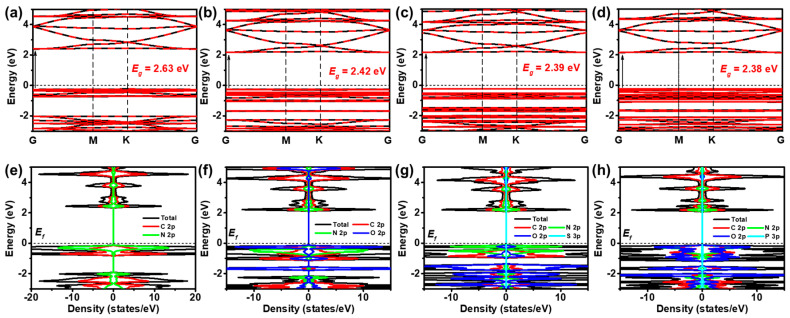
Band structure of (**a**) CTF, (**b**) CTF-COOH, (**c**) CTF-HSO_3_, and (**d**) CTF-H_2_PO_3_. The density of states (DOS) of (**e**) CTF, (**f**) CTF-COOH, (**g**) CTF-HSO_3_, and (**h**) CTF-H_2_PO_3_.

As depicted in Figure 3a–d, the calculated band gaps for CTF, CTF-COOH, CTF-HSO_3_, and CTF-H_2_PO_3_ are 2.63, 2.42, 2.39, and 2.38 eV, respectively. Notably, CTF-H_2_PO_3_ exhibits the smallest band gap among these materials. A comparison of the absorption edges of CTF highlights that the theoretically determined band gaps align well with the optical absorption characteristics of these materials. Like pristine CTF, the functionalized derivatives CTF-COOH, CTF-HSO_3_, and CTF-H_2_PO_3_ all exhibit direct band gaps. This indicates that the addition of functional groups does not alter the semiconducting nature of the band gap, preserving the high optical absorption efficiency and robust photogenerated carrier separation. In a direct band gap semiconductor, electrons transition from the valence band (VB) to the conduction band (CB) without a change in momentum, significantly enhancing photon absorption efficiency, particularly in the UV–Vis light regions. Further analysis of the DOS reveals distinct orbital contributions (Figure 3e–h). In pristine CTF, the VB is primarily composed of the N 2p orbitals, while the CB is largely derived from the C and N 2p orbitals. This disparity is attributed to the higher electronegativity of N, which favors electron acceptance, making the N 2p orbitals dominant in the VB. This phenomenon can be linked to the conjugated π-bond structure of TR, where π-electron clouds are delocalized between N and C atoms. Interestingly, when additional functional groups such as -H_2_PO_3_ are introduced to form CTF-H_2_PO_3_, the electronic structure undergoes significant modification. The VB shifts from being dominated by the N 2p orbitals to the O 2p orbitals.

**Figure 4:**
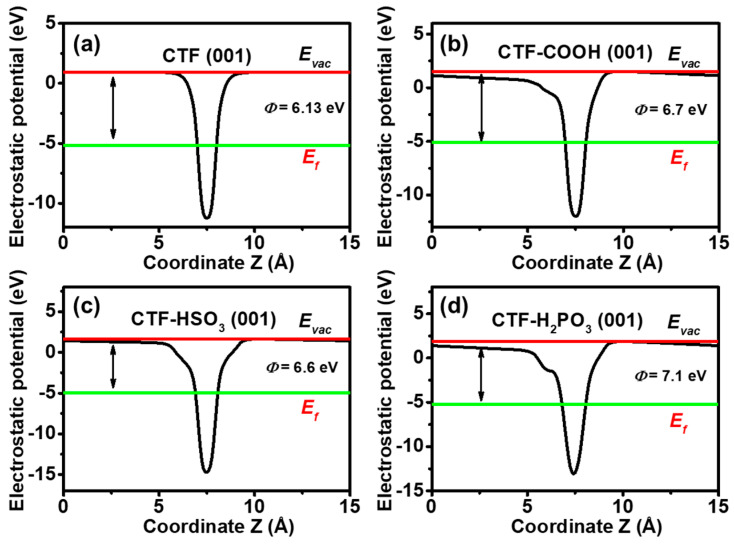
Electrostatic potentials curves of the (**a**) CTF, (**b**) CTF-COOH, (**c**) CTF-HSO_3_, and (**d**) CTF-H_2_PO_3_.

To analyze the potential of CTF-based materials for photocatalytic degradation of pesticide contaminants such as dichlorvos (DDVP), the work function (W_f_) of the CTF (001) crystal face was calculated (Figure 4a–c). The W_f_ for pristine CTF was found to be 6.13 eV (Figure 4a). Introducing electron-withdrawing functional groups like -COOH, -HSO_3_, and -H_2_PO_3_ significantly alters the electronic properties of CTFs. These groups act as electron acceptors, lowering the Fermi levels of the modified CTFs (Figure 4a–d). Consequently, the W_f_ increased to 6.70, 6.60, and 7.10 eV for CTF-COOH, CTF-HSO_3_, and CTF-H_2_PO_3_, respectively. A larger work function indicates stronger electron confinement by the catalyst. The introduction of H_2_PO_3_ results in the greatest increase in electronegativity, suggesting that H_2_PO_3_ has the strongest electron-binding ability. For donor-acceptor CTFs, the electron donor typically forms the major component of the conduction band, while the electron acceptor is generally part of the valence band [23]. Therefore, the trend of an increasing work function also suggests that these oxygen-containing acid functional groups primarily contribute to the valence band. This is consistent with the analysis of the band structure and density of states.

**Figure 5:**
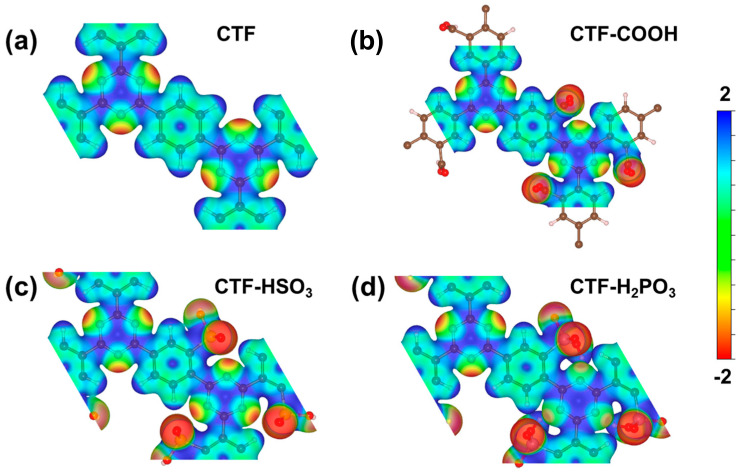
Surface electrostatic potential of (**a**) CTF, (**b**) CTF-COOH, (**c**) CTF-HSO_3_, and (**d**) CTF-H_2_PO_3_.

To determine the effect of oxygen-containing acid groups on the surface distribution of potential and active sites, the surface electrostatic potential of the CTFs before and after modification was simulated. Figure 5a–d presents the top-view distribution of surface electrostatic potentials for various CTF variants, offering a visual depiction of how distinct electron-accepting functional groups influence the electrostatic potential. The results highlight the varying degrees of electron redistribution caused by these functional groups, which directly affect the surface properties of the materials. The red regions have a lower electrostatic potential, while the blue regions have a higher electrostatic potential. For CTF, the electrostatic potential at the N sites is lower, while the potential at the H and C sites is higher. This indicates that the surface electrons of CTF are primarily concentrated at the N sites of the triazine rings, which is consistent with the electronic structure and work function analysis. Under light irradiation, photo-induced electrons in the red regions will transfer to the blue regions, causing the N sites to lose electrons and thereby retain more photo-induced holes. Consequently, the N sites act as oxidative active sites, participating in the oxidation of DDVP. In contrast to pure CTF, after the introduction of oxygen-containing acid groups, these groups become the regions with the lowest electrostatic potential and the highest electron density. Furthermore, the electron density and spatial distribution of these electron-rich regions are much larger than those at the N sites of pure CTF. This suggests that these functional groups not only serve as oxidation sites but also exhibit significantly stronger oxidative capabilities compared to pure CTF. Notably, the H_2_PO_3_ sites have the lowest potential and the largest effective range, indicating that H_2_PO_3_ can exert a stronger oxidative effect and degradation efficiency on DDVP during photodegradation processes.

**Figure 6:**
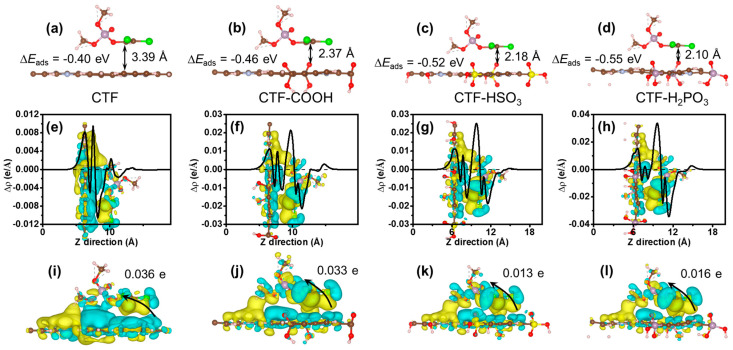
Interaction between DDVP and (**a**) CTF, (**b**) CTF-COOH, (**c**) CTF-HSO_3_, and (**d**) CTF-H_2_PO_3_. Charge density difference and charge transfer of (**e**,**i**) CTF, (**f**,**j**) CTF-COOH, (**g**,**k**) CTF-HSO_3_, (**h**,**l**) CTF-H_2_PO_3_.

Through a detailed analysis of molecular configuration, charge distribution, and electronic transfer properties, the modification mechanism of oxygen-containing acid functional groups on the performance of CTF was elucidated. As shown in Figure 6a, the adsorption distance between unmodified CTF and the DDVP molecule is 3.39 Å. After introducing functional groups, the adsorption distances were reduced to 2.37, 2.18, and 2.10 Å, respectively (Figure 6b–d). This reduction in distance can be attributed to the enhanced interaction between the CTF surface and the DDVP molecule due to the incorporation of oxygen-containing acid functional groups, thereby improving the adsorption capacity. The adsorption energy (∆E_ads_) of unmodified CTF was calculated to be −0.40 eV, while the introduction of oxygen-containing acid functional groups increased the adsorption energy to −0.46, −0.52, and −0.55 eV, respectively. These results indicate that the inclusion of functional groups significantly enhances the adsorption ability of CTF toward DDVP, primarily due to stronger electrostatic interactions, particularly van der Waals forces. Figure 6e–h present the differential charge density distributions after DDVP adsorption, where yellow regions represent electron accumulation and blue regions indicate electron depletion. These plots reveal minimal charge fluctuations between the two molecules, indicating that there is mutual electron transfer between DDVP and CTFs, which is one reason for the relatively low total interfacial electron transfer. To better determine the direction and electron transfer quantity, the electron gain and loss between DDVP and CTFs were analyzed using Bader charge (Figure 6i–l). Specifically, unmodified CTF exhibits a charge transfer of 0.036 e from CTFs to DDVP. After electron transfer, an electrostatic field is generated from CTFs towards DDVP. Under light irradiation, this electric field drives the photo-induced holes to transfer to DDVP, thereby facilitating its rapid degradation. The inclusion of COOH, HSO_3_, and H_2_PO_3_ functional groups adjusts the charge transfer to 0.033, 0.013, and 0.016 e, respectively. This discrepancy between the electron transfer amount and adsorption energy may be because the adsorption energy is more influenced by the interaction distance, while the electron transfer is less dependent on it. Overall, these findings provide deeper insights into the role of oxygen-containing acid functional groups in enhancing the adsorption performance and oxidation behavior of CTFs.

**Figure 7:**
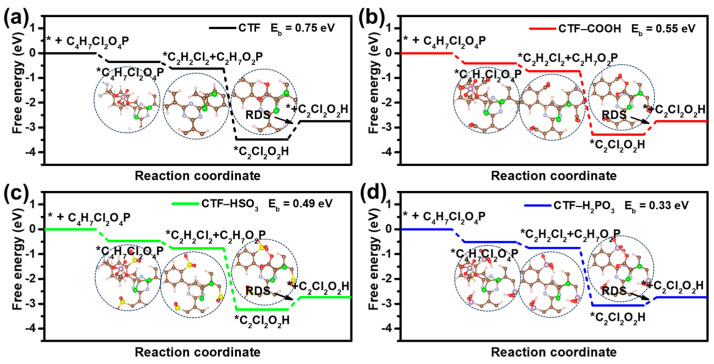
Free-energy diagrams for the photodegradation and reaction path of DDVP of (**a**) CTF, (**b**) CTF-COOH, (**c**) CTF-HSO_3_, and (**d**) CTF-H_2_PO_3_.

To further explore the intermediate transformation during DDVP degradation on CTF, DFT was employed to assess the free energy changes associated with the oxidation of DDVP (C_2_H_7_Cl_2_O_4_P) to dichloroacetic acid (DCA, C_2_Cl_2_O_2_H) on CTF (Figure 7a–d). Initially, DDVP adsorbs onto the TR active site of CTF, forming an adsorbed species (Equation (1)). This step is spontaneous and requires no energy input. Similarly, for CTF-COOH, CTF-HSO_3_, and CTF-H_2_PO_3_, the initial adsorption process remains spontaneous, with modified CTFs showing more significant reductions in free energy, suggesting the process becomes more favorable. In the subsequent step, the adsorbed DDVP transforms into adsorbed 1,2-dichloroethane (DCE, C_2_H_2_Cl) and dimethyl phosphoric acid (DPA, C_2_H_7_Cl_2_P) (Equation (2)), which is also a spontaneous reaction. This transformation remains spontaneous on CTF-COOH, CTF-HSO_3_, and CTF-H_2_PO_3_, with notable free energy reductions, indicating that the reaction is more kinetically favorable. The next step involves the conversion of *DCE into *DCA (Equation (3)), which is also spontaneous. In the final step (Equation (4)), *DCA desorbs from the TR site of CTF. Unlike the earlier steps, this transformation is non-spontaneous and requires additional energy input. The rate-determining step (RDS) of the entire degradation process is the desorption of *DCA from the TR site on CTF (Equation (4)), with the energy barrier representing the critical bottleneck of the reaction. The reaction barriers for CTF, CTF-COOH, CTF-HSO_3_, and CTF-H_2_PO_3_ are 0.75, 0.55, 0.45, and 0.33 eV, respectively. Compared with unmodified CTF, the reaction barrier of the modified CTFs is significantly reduced, primarily due to the electrostatic repulsion between the oxygen atoms in the DCA molecule and those in the modified functional groups. Additionally, CTF-H_2_PO_3_, with the lowest energy barrier (0.33 eV) among the four catalysts, demonstrates excellent DDVP degradation performance.

(1) $$*+\;C_{4}H_{7}{Cl}_{2}O_{4}P\to \;*C_{4}H_{7}{Cl}_{2}O_{4}P\;.$$

(2) $$*C_{2}H_{7}{Cl}_{2}O_{4}P\to \;*C_{2}H_{2}{Cl}_{2}+C_{2}H_{7}O_{2}P.$$

(3) $$*C_{2}H_{2}{Cl}_{2}\to *C_{2}{Cl}_{2}O_{2}H.$$

(4) $$*C_{2}{Cl}_{2}O_{2}H\to *\;C_{2}{Cl}_{2}O_{2}H$$

## 4. Conclusions

This study systematically investigated the mechanism of photodegradation of DDVP using oxygen-containing acid functional CTFs. The results demonstrate that the introduction of -COOH, -HSO_3_, and -H_2_PO_3_ functional groups offers several key advantages: (1) narrowing the band gap of CTFs, thereby promoting visible light absorption; (2) enhancing the dipole moment, which facilitates charge separation; (3) serving as the main component of the valence band and acting as oxidative active sites; (4) improving antioxidant capacity, thereby enhancing the stability of the photocatalytic degradation process; (5) strengthening the adsorption and activation of DDVP, promoting the desorption of acidic oxygenated products, and lowering the reaction energy barrier. Among them, the -H_2_PO_3_ group markedly reduces the reaction energy barrier, enhancing the activation capability and oxidation durability of CTFs, thereby demonstrating significant potential in the photodegradation of DDVP. This research presents valuable insights into the development of efficient, stable, and environmentally friendly photocatalysts for pesticide degradation.

## Data Availability

Data are contained within the article.

## References

[1] Mei L., Xia X., Cao J., Zhao Y., Huang H., Li Y., Zhang Z. (2024). Degradation of Three Herbicides and Effect on Bacterial Communities under Combined Pollution. Toxics.

[2] Do K.H., Kumar D.P., Rangappa A.P., Lee J., Yun S., Kim T.K. (2023). Design and synthesis of a covalent organic framework bridging CdS nanoparticles and a homogeneous cobalt–bipyridine cocatalyst for a highly efficient photocatalytic CO_2_ reduction. J. Mater. Chem. A.

[3] Yi Y., Guan Q., Wang W., Jian S., Li H., Wu L., Zhang H., Jiang C. (2023). Recyclable Carbon Cloth-Supported ZnO@Ag_3_PO_4_ Core-Shell Structure for Photocatalytic Degradation of Organic Dye. Toxics.

[4] Lotfi S., Ouardi M.E., Ahsaine H.A., Assani A. (2022). Recent progress on the synthesis, morphology and photocatalytic dye degradation of BiVO_4_ photocatalysts: A review. Catal. Rev..

[5] Wu X., Tan L., Chen G., Kang J., Wang G. (2024). g-C_3_N_4_-based S-scheme heterojunction photocatalysts. Sci. China Mater..

[6] Fragoso J., Barreca D., Bigiani L., Gasparotto A., Sada C., Lebedev O.I., Modin E., Pavlovic I., Sánchez L., Maccato C. (2022). Enhanced photocatalytic removal of NO_x_ gases by β-Fe_2_O_3_/CuO and β-Fe_2_O_3_/WO_3_ nanoheterostructures. Chem. Eng. J..

[7] Yue W., Wang X., Zhang J., Bao J., Yao M. (2024). Degradation Characteristics of Nicosulfuron in Water and Soil by MnO_2_ Nano-Immobilized Laccase. Toxics.

[8] Shen L., Ye T., Chen Y., Chu B., Chen H., Hu J., Yu Y. (2024). Facile Synthesis of a Novel AgIO_3_/CTF Heterojunction and Its Adsorption-Photocatalytic Performance with Organic Pollutants. Toxics.

[9] Kurisingal J.F., Kim H., Choe J.H., Hong C.S. (2022). Covalent organic framework-based catalysts for efficient CO_2_ utilization reactions. Coord. Chem. Rev..

[10] Ding H., Wang Y.-R., Liu M., Shi J.-W., Yu T.-Y., Xia Y.-S., Lu M., Yang Y.-L., Chen Y., Li S.-L. (2022). Electronic Tuning of Active Sites in Bifunctional Covalent Organic Frameworks for Photoassisted CO_2_ Electrocatalytic Full Reaction. Chem. Mater..

[11] Lu Z., Wang Z. (2024). Complete Photooxidation of Formaldehyde to CO_2_ via Ni-Dual-Atom Decorated Crystalline Triazine Frameworks: A DFT Study. Toxics.

[12] Nguyen H.L., Alzamly A. (2021). Covalent Organic Frameworks as Emerging Platforms for CO_2_ Photoreduction. ACS Catal..

[13] Kim Y.H., Kim N., Seo J.-M., Jeon J.-P., Noh H.-J., Kweon D.H., Ryu J., Baek J.-B. (2021). Benzothiazole-Based Covalent Organic Frameworks with Different Symmetrical Combinations for Photocatalytic CO_2_ Conversion. Chem. Mater..

[14] Hou Y., Lin S., Fan J., Zhang Y., Jing G., Cai C. (2024). Enhanced Adsorption of Cadmium by a Covalent Organic Framework-Modified Biochar in Aqueous Solution. Toxics.

[15] Chen G., Zheng Z., Zhong W., Wang G., Wu X. (2024). Molten Intermediate Transportation-Oriented Synthesis of Amino-Rich g-C_3_N_c_ Nanosheets for Efficient Photocatalytic H_2_O_2_ Production. Acta Phys. Chim. Sin..

[16] Lu Z., Lv H., Liu Q., Wang Z. (2024). Modulating NH_2_ Lewis Basicity in CTF-NH_2_ through Donor-Acceptor Groups for Optimizing Photocatalytic Water Splitting. Acta Phys. Chim. Sin..

[17] Lu T., Chen Q. (2021). Shermo: A general code for calculating molecular thermochemistry properties. Comput. Theor. Chem..

[18] Lu T., Chen F. (2012). Multiwfn: A multifunctional wavefunction analyzer. J. Comput. Chem..

[19] Zhou Q., Guo Y., Zhu Y. (2023). Photocatalytic sacrificial H2 evolution dominated by micropore-confined exciton transfer in hydrogen-bonded organic frameworks. Nat. Catal..

[20] Xiong X.H., Yu Z.W., Gong L.L., Tao Y., Gao Z., Wang L., Yin W.H., Yang L.X., Luo F. (2019). Ammoniating Covalent Organic Framework (COF) for High-Performance and Selective Extraction of Toxic and Radioactive Uranium Ions. Adv. Sci..

[21] Pang X., Shi B., Liu Y., Li Y., Zhang Y., Wang T., Xu S., Wang X., Liu Z., Xing N. (2024). Phosphorylated Covalent Organic Framework Membranes Toward Ultrafast Single Lithium-Ion Transport. Adv. Mater..

[22] Li J.F., Zhang Y.J., Ding S.Y., Panneerselvam R., Tian Z.Q. (2017). Core-Shell Nanoparticle-Enhanced Raman Spectroscopy. Chem. Rev..

[23] Lu Z., Xu X., Wang Z. (2024). Highly crystalline carbon nitride by covalent remedy for CO_2_ photoreduction. Sep. Purif. Technol..

